# Natural selection, selective breeding, and the evolution of resistance of honeybees (*Apis mellifera)* against *Varroa*

**DOI:** 10.1186/s40851-020-00158-4

**Published:** 2020-05-18

**Authors:** Jacques J. M. van Alphen, Bart Jan Fernhout

**Affiliations:** 1 Naturalis Biodiversity Centre, 2333 CR Leiden, The Netherlands; 2 Arista Bee Research Foundation, Nachtegaal 2, 5831 WL Boxmeer, The Netherlands

**Keywords:** Honeybee, *Varroa destructor*, Resistance, Virus tolerance, Natural selection, Selective breeding, Hygienic behaviour, Grooming, Recapping

## Abstract

We examine evidence for natural selection resulting in *Apis mellifera* becoming tolerant or resistant to *Varroa* mites in different bee populations. We discuss traits implicated in *Varroa* resistance and how they can be measured. We show that some of the measurements used are ambiguous, as they measure a combination of traits. In addition to behavioural traits, such as removal of infested pupae, grooming to remove mites from bees or larval odours, small colony size, frequent swarming, and smaller brood cell size may also help to reduce reproductive rates of *Varroa*. Finally, bees may be tolerant of high *Varroa* infections when they are resistant or tolerant to viruses implicated in colony collapse. We provide evidence that honeybees are an extremely outbreeding species. Mating structure is important for how natural selection operates. Evidence for successful natural selection of resistance traits against *Varroa* comes from South Africa and from Africanized honeybees in South America. Initially, *Varroa* was present in high densities and killed about 30% of the colonies, but soon after its spread, numbers per hive decreased and colonies survived without treatment. This shows that natural selection can result in resistance in large panmictic populations when a large proportion of the population survives the initial *Varroa* invasion. Natural selection in Europe and North America has not resulted in large-scale resistance. Upon arrival of *Varroa*, the frequency of traits to counter mites and associated viruses in European honey bees was low. This forced beekeepers to protect bees by chemical treatment, hampering natural selection. In a Swedish experiment on natural selection in an isolated mating population, only 7% of the colonies survived, resulting in strong inbreeding. Other experiments with untreated, surviving colonies failed because outbreeding counteracted the effects of selection. If loss of genetic variation is prevented, colony level selection in closed mating populations can proceed more easily, as natural selection is not counteracted by the dispersal of resistance genes. In large panmictic populations, selective breeding can be used to increase the level of resistance to a threshold level at which natural selection can be expected to take over.

## Introduction

*Varroa destructor* (Anderson and Truman, 2000) is an external parasitic mite of honeybees that shifted from its original host *Apis cerana*, the Asian hive bee, to *Apis mellifera*, the European honeybee. In the mid 1800s, settlers from western Russia carried *A. mellifera* to the far southeastern corner of Russia, into an area where *A. cerana* occurs naturally [1]. Contact between the two species resulted in the introduction of *Varroa* to *A. mellifera*. The *Varroa* mite arrived in Moscow during the 1950s with honeybees from the east. The parasite spread rapidly and colonized western Europe and North America in the early 1980s, and since its invasion from Russia, Varroa has been the major mortality factor of honeybees. *Varroa* mites are vectors of several bee viruses, and at high mite densities these viruses cause colony collapse [2]. While it does not cause colony mortality in its original host *A. cerana* [3, 4], it became and remains devastating to apiculture and to natural populations of *A. mellifera* in Europe and feral populations in North America. One reason why *Varroa* is so virulent on *A. mellifera* is that it can breed in worker brood and so achieve a long reproductive season, while in *A. cerana,* pupae from worker cells with reproducing mites are always removed [5] and breeding is restricted to the short season during which drones are produced.

*Varroa* has been present in European and American *A. mellifera* populations for almost 40 years and, as an important mortality factor, it presumably exerts strong natural selection for resistance in these populations. We call a bee colony resistant when it is able to limit the population size of *Varroa*, to a density that does not cause mortality.

In western Europe and North America, hives are frequently treated with acaricides, natural acids, or essential oils to control *Varroa*, and *Varroa* reproduction is disrupted by other apicultural measures [6]. Moreover, a large proportion of the hives are regularly requeened. These practices are thought to hamper natural selection for resistance against *Varroa.* However, not all beekeepers treat their colonies against *Varroa*, and in Europe, where *A. mellifera* is native, wild colonies of honey bees were not uncommon when *Varroa* arrived [7]. Likewise, populations of feral colonies of honeybees in North America are exposed to natural selection. Evidence for small-scale natural selection from some of these untreated colonies provides insight into why natural selection in the European honeybee has not resulted in population-wide resistance.

### Traits contributing to *Varroa* resistance

#### Varroa *Resistance in A. cerana*

The mite stably coexists with its original host. *Apis cerana* workers prevent the growth of *V. destructor* populations by different behavioural traits known as ‘grooming’, ‘uncapping and removing’ and ‘entombing’ [4, 8]. *Apis cerana* bees groom themselves (‘auto-grooming’) and also perform grooming dances to recruit nestmates to engage in social grooming (‘allo-grooming’). This often results in the removal of phoretic adult mites and inflicts significant mortality among them. The uncapping of worker cells with reproducing *Varroa* and the subsequent removal of the parasitized pupae result in the removal of mite offspring before they have been able to reproduce [5]. This is an important factor in preventing the mite population to grow to harmful densities. Of *Varroa* females that enter a worker cell, 90% do not reproduce, which could be caused by a so-called “brood effect”, i.e. the suppression of *Varroa* reproduction by the brood. The few that do lay eggs fail because of the uncapping and removing behaviour. In *A. cerana*, *Varroa* only reproduces successfully in drone cells. In drone cells that have been colonized by two or more adult females, the host often dies [4]. *Apis cerana* workers leave the dead drone brood capped, thus entombing the reproducing parasites and their offspring and causing mortality of up to 25% among the mites [4]. Drones are produced during a relatively short season [9, 10], allowing the mites to produce only 3–5 generations per year. This is one of the reasons why *Varroa* is an innocuous parasite of *A. cerana*.

#### Traits for Varroa resistance in *A. mellifera*

**Fig. 1:**
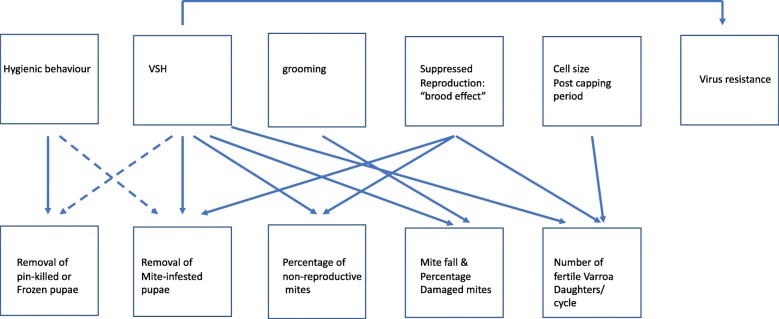
Relationships among honeybee resistance traits and data collected to measure them. Arrows explained in the text

Three of the traits that provide resistance against *Varroa* in *A. cerana*, are also present in European *A. mellifera* populations*,* albeit in low frequency: the uncapping of *Varroa*-infected cells and subsequent removal of parasitized pupae, as well as auto- and allo-grooming [11]. Another trait that might confer resistance against *Varroa* is the “brood effect”. The mechanism for the suppression of mite reproduction is a change in a chemical signal issued by a developing worker larva that, if unaltered, would be used by *Varroa* mites as a signal to initiate reproduction [12, 13]. Entombing has not been observed in *A. mellifera*. We first review the evidence for the traits that confer or are thought to confer resistance. We show that evidence cited in support of a particular resistance trait could sometimes also be produced by one of the other traits (Fig. 1). Next, we review the evidence that natural selection has resulted in resistance against *Varroa* in *A. mellifera* in Africa, Europe, and the Americas, and the roles played by the different resistance traits.

Uncapping of *Varroa*-infested cells and removal of parasitized pupae.

### Hygienic behaviour

Hygienic behaviour was discovered by Park and co-workers [14]. They observed that bees in colonies that appeared to be resistant against American foulbrood removed larvae from cells inoculated with the disease. Their results indicated that the behaviour was heritable. Woodrow & Holst [15] labelled this activity “hygienic behaviour” and provided further evidence that resistance to American foulbrood in a honeybee colony consists in its ability to detect and remove diseased brood before the causative organism, *B. larvae*, reaches the infectious spore stage in the diseased larvae. Rothenbuhler [16] studied the genetics of hygienic behaviour and provided evidence that it is compatible with two recessive genes coding for the behaviour. Later research [17, 18] found evidence that more genes are involved, which is not surprising for a behavioural trait. Indeed, Harpur et al. [19] found 73 candidate genes associated with hygienic behaviour. Most of those genes play roles in regulating the expression of genes involved in neuronal development, which could explain ﻿why hygienic bees are more receptive to olfactory signals associated with dead brood.

Woodrow & States [20] already suggested that hygienic behaviour was not specific to American foulbrood: “ … it is likely that the removal of diseased brood is a common behaviour of bees” (cited in [21]. Indeed, subsequent studies [22, 23] showed that adult bees from some colonies also removed larvae infected with chalk brood from their cells within 24 h, and that this behaviour also plays an important role in the resistance of bees against this disease [24, 25]. Based on the belief that the hygienic response to diseased and dead brood is general, a test for hygienic behaviour using freeze-killed brood was designed [23, 26, 27].

The belief that the hygienic behaviour is a general response to diseased and dead brood also led to the hypothesis that hygienic behaviour could play a role in resistance against *Varroa*. This hypothesis was tested by Boecking & Drescher [28] and Spivak [29]. ﻿Boecking and Drescher [28] found a positive correlation (r = 0.74) between the removal of brood infested with two mites per cell and the removal of freeze-killed brood, suggesting that the hygienic response to *Varroa*-infected cells can at least in part be explained by the general response of hygienic bees to dead and diseased larvae. Spivak [29] repeated and extended these experiments with similar, albeit variable results.

Hence, hygienic bees selected for removing dead larvae show a higher incidence of removing larvae from cells infested with *Varroa* than non-hygienic control bees (Fig. 1). However, in Boecking & Drescher’s [28] study, this only explained 55% of the variance. Danka et al. [30] also found only a weak relation between the removal of dead brood and VSH behaviour. Likewise, Boecking et al. [31] showed that the rate of removal of pin-killed larvae correlates with the rate of removal of *Varroa* infested cells, but that this accounted for only 37% of the variance. They termed the removal of larvae from *Varroa* infested cells as “*Varroa*-specific hygiene”, and were first in recognizing that the genetic background of bees removing pupae from *Varroa*-infested cells is different from bees that only remove killed pupae.

### Varroa-sensitive hygiene

Evidently, apart from general hygienic behaviour, other traits must be involved in the removal of pupae from *Varroa* infested cells. The cues bees use to detect and remove frozen or pin-killed pupae are not necessarily the same as those used ﻿to detect and remove mite-infested pupae. Harbo & Hoopingarner [32] looked for heritable resistance of honeybees without limiting themselves to hygienic behaviour. As natural mating of bee queens results in queens inseminated by multiple males, they used queens artificially inseminated with sperm of only a single male to ensure that resistance characteristics could be strongly expressed by a whole colony. Experimental colonies were infested with equal densities of mites and 63 or 70 days later the mite population size in the colonies was determined. In addition, they measured the incidence of hygienic behaviour using sections of frozen brood, determined the proportion of damaged fallen mites as a measure for grooming behaviour, the incidence of non-reproduction of mites that were found inside capped cells, and duration of the capped period as a measure for developmental time. Of these traits, only the percentage non-reproduction showed a negative correlation with the increase in the mite population that explained 53% of the variance. They concluded that suppressed mite reproduction (SMR) could be an important resistance trait against *Varroa*.

In a later study, Harbo and Harris [33] measured the heritability of potential traits for resistance and found that suppression of mite reproduction, hygienic behaviour and proportion of mites in brood cells were candidates for selection due to high heritability. They then started a programme for the selection of bees with the ability to suppress reproduction in the mites, that after about five generations of selection produced colonies that had < 6% of their mites classified as reproductive in worker cells [34]. Ibrahim & Spivak [35] hypothesized that the selective removal of brood infested with reproductive mites could explain the observed decrease in percentage reproductive mites. Harbo & Harris [34] tested this hypothesis. They placed frames with newly capped worker brood in SMR and in control colonies and counted *Varroa*-infested cells and measured the proportion of reproducing mites after 7–9 days. They found that in SMR colonies the percentage of infested cells had been reduced from 22 to 9%, and the percentage of infested cells containing reproducing mites had decreased from 71 to 20%. The number of cells containing non-reproducing mites was not different between SMR and control colonies. These results show that in the SMR colonies, cells containing reproducing mites had disappeared, and cells containing non-reproducing mites had been left untouched. This is evidence that SMR bees show hygienic behaviour preferentially to cells with reproducing mites. Hence, this particular form of hygienic behaviour is causing an increase in the percentage of non-reproducing mites by reducing the proportion of reproducing mites (Fig. 1).

Harbo & Harris [36] and Harris, [37], Harris et al. [38] and Kim et al. [39] confirmed these findings. Harris [37] renamed SMR and called the behaviour of uncapping cells containing reproducing *Varroa* and the removal of pupae from such cells “*Varroa* Sensitive Hygiene” (VSH), a process that results in the removal of mite offspring before they are able to reproduce successfully, thus interrupting the reproductive cycle of the *Varroa.* VSH behaviour is a heritable trait that responds well to selection [33]. Bees with VSH recognize cells containing reproducing *Varroa* [12, 13, 40]. There are indications that at least 2 major genes with additive effects are involved [41]. Tsuruda et al. [42] did a QTL analysis for VSH and found two QTL’s, one on chromosome 9 and one on chromosome 1, together explaining only 10% of the variance. On average, individuals that were homozygous for the VSH allele were more likely to be individuals who were observed exhibiting VSH. Spöter et al. [43] found six candidate genes and Scannapieco et al. [44] found five genes associated with VSH behaviour. It is likely that some of the genes involved in hygienic behaviour also play a role in VSH.

The proportion of workers in a colony expressing VSH behaviour is positively correlated with the proportion of non-reproducing mites in the brood [34, 36]. This is because VSH bees preferentially attack cells with reproducing mites [34, 38].

A low frequency of VSH behaviour must be present in almost every population of European honeybees in Europe and North America, because it has been found when looked for. (e.g. [30]: low VSH in commercial control colonies) [32, 45–48].

### Recapping

In experiments to measure VSH it has often been observed that pupae infested with *Varroa* that had been uncapped were recapped without the host pupa being injured [30, 31, 49–54]. Although the foundress mite may escape an uncapped brood cell before it is recapped, she usually remains within the cell [31, 50]. Brood exposed for one week to bees selected for VSH often have high mean percentages (> 30%) of recapped brood cells [54, 55], and some ﻿colonies may have > 90% of all brood recapped. Most of these recapped cells are not infested by *Varroa*, but about 20% of recapped cells contain a mite [55]. Martin et al. [56] consider recapping as the repair of cells erroneously opened by VSH bees.

Harris et al. [57] suggested that it is possible that hygienic uncapping followed by recapping of brood cells could inhibit or alter mite reproduction. This would be an alternative explanation for the increased percentage of non-reproductive mites found by Harbo & Harris [34, 36], who did not discriminate between normally capped and recapped cells. This hypothesis was tested by Harris et al. [38]. They found that ﻿the frequency of pupae with remaining fertile mites in normally capped brood cells for control bees was 10 × that found for VSH bees, confirming Harbo & Harris [34, 38] conclusion that VSH bees preferentially target cells with reproducing mites. ﻿The total number of offspring for fertile mites did not vary between normal and recapped cells, indicating that offspring were not removed before cells were recapped. Mortality of mite offspring was significantly higher in recapped cells than in normally capped cells and contributed to decreased reproduction by the mites [38].

Harris et al. [38, 57] considered recapping to be part of VSH behaviour. However, in the colonies labelled as VSH, not all bees exhibited VSH behaviour, as part of the workers were from patrilines not expressing VSH. Therefore, it could also have been non-VSH bees that recapped the cells opened by de VSH bees. Evidence that non-hygienic bees recap cells comes from Spivak & Gilliam [24]. When they added young non-hygienic bees to hygienic colonies, hygienic behaviour was suppressed. In a different experiment, they showed that non-hygienic bees tended to recap partially uncapped cells containing dead brood, whereas hygienic bees never recapped those cells. De Guzman et al. [58] found higher recapping rates in Italian bees with low rates of VSH behaviour, than in the resistant Russian honeybees that displayed high rates of VHS. More evidence comes from Boecking & Spivak [49], who found that bees from pre-selected non-hygienic colonies tended to recap partially uncapped cells that contained freeze-killed brood and from Arathi et al. [52] who demonstrated that in mixed colonies, as compared to a colony of hygienic bees, a higher proportion of uncapped cells were subsequently recapped, resulting in delayed removal of dead brood.

Hence, recapping by non-hygienic bees appears to counteract the activity of the hygienic workers and probably reduces the efficacy of hygienic behaviour against *Varroa*. More information is needed to know how this depends on the ratio between non-hygienic and VSH-bees in a colony.

### Measuring Varroa-sensitive hygiene

Many studies have used frozen or pin-killed brood to assess the rate of removal of pupae infested with *Varroa*. These assays measure hygienic behaviour, which, as we have seen, correlates only partly with VSH behaviour. Therefore, VSH should be measured using brood infested with *Varroa*.

### Grooming

#### Auto- and allogrooming

As in other insects, adult bees clean their bodies by grooming themselves (“auto-grooming”). As mentioned above, they may also perform a grooming dance to solicit grooming by other bees. Grooming by other bees is called “allo-grooming”. This behaviour has been described in detail by Land & Seeley [59]. Auto and allo-grooming are performed by all honeybees to remove dust or pollen. Auto- and allo-grooming in response to the presence of a *Varroa* mite play important roles in the resistance of *A. cerana* against *Varroa* [60–62]. In *A. cerana* hives, 73.8% of fallen mites exhibited damage [60]. The evidence is based on direct observation of the grooming behaviour in combination with data on the rate of successful mite removal from bees, the percentage of mites dropping to the bottom board and the percentage of damaged mites. It points to a direct relationship between grooming behaviour and the falling of mites to the bottom board. The *Varroa* mites in the Peng et al. [60] publication originated from *A. mellifera*. This may have resulted in a higher percentage of damaged mites than if the *A. cerana* bees had been tested with mites from conspecifics.

#### Incidence of grooming against Varroa in *A. mellifera*

Grooming behaviour against *Varroa* in *A. mellifera* is expressed at a lower frequency and intensity than in *A. cerana* [8, 62]. Depending on subspecies or on differences between colonies of the same subspecies *A. mellifera* adults vary in response to being mounted by a *Varroa* mite. They may or may not react by auto-grooming or by performing the invitation-to-grooming dance. *A. mellifera* bees from colonies that are resistant to *Varroa* show more vigorous and more effective grooming responses [63, 64], but good data on the relation between grooming and mite mortality are not available.

#### Measuring the incidence of grooming behaviour

Grooming against *Varroa* mites has been associated with higher proportions of mutilated mites falling from bees in colonies [65] and the proportion of mutilated mites was associated with lower infestation levels [65–68]. Moosbeckhofer [67] found a significant negative correlation between the proportion of dark damaged fallen mites and brood infestation rates. *Apis mellifera* colonies with the lowest rate of mite population growth showed more grooming behaviour, more mites falling to the hive floor, higher proportions of fallen chewed mites, and reduced infestation levels of adult bees [65]. These observations have been the basis for using the proportion of damaged mites as a measure for grooming behaviour and the belief that it may be a useful parameter in selecting for *Varroa* resistance.

The proportion of damaged mites fallen to the hive floor varies greatly between colonies and between subspecies: Colonies of *A. m. ligustica* showed an average mite damage rate of 5.75%. In contrast, Rosenkranz et al. [69], working with *A. m. ligustica* and *A.m. carnica*, recorded mite damage rates averaging 45% (44–62%), while Africanized *A. mellifera* damaged 38.5% [70]. Ruttner [71] and Ruttner and Hänel [66] provided evidence for active defence of some surviving colonies of *A. m. carnica* against phoretic *Varroa*, based on the observation of fallen mites with damaged legs and cuticle of the dorsal shield, or idiosoma. Later it was reported [72] that these strong *A. m. carnica* hives eventually achieved a damage rate of 93%. *Apis m. mellifera* has not been as well studied as the above-mentioned subspecies, but a Polish population of *A. m. mellifera* bees was much more reactive to *Varroa* mites than bees from local populations of *A. m. carnica* and *A. m. caucasica*: 98% showing some response to contact with a mite [73].

The question is whether the variation in the proportion of damaged mites reflects heritable variation in grooming behaviour against *Varroa* mites. Moosbeckhofer [67] reported that 53.7% of the mites that had fallen to the bottom in the *A. m carnica* colonies he studied were light-coloured young females, 27.1% of which were damaged. Such light-coloured females may well have originated from cells after eclosion of a young parasitized worker bee. However, they could as well have originated from cells opened by workers showing VSH behaviour and have been removed with the parasitized pupa or during tidying up of the cell after removal of the pupa. Supporting evidence for the latter scenario comes from Hoffman ﻿ [74], who assessed damage rates in fallen mites in relation to the development of the brood nest. When no brood was emerging, the damage rate was 10.2%, but was significantly higher at 16.7%, with emerging brood, when the multiple injury rate of mites was also higher. Lobb and Martin [75] estimated that around 50% of fallen dead mites die within sealed brood cells, the rest mainly shortly after emergence. Martin ﻿ [76] reported that numbers of fallen mites increased by a factor of 6 [75], or 7–15 [77] when *A. mellifera* brood was emerging, compared to when it was not. Rosenkranz et al. [69] monitored the proportion of damaged mites in the floor debris of *A. mellifera* hives with and without emerging brood and when *Varroa*-sensitive hygiene was stimulated by the insertion of newly killed, but otherwise intact *Varroa* mites. They found that dead mites removed from brood cells by the bees were damaged to a similar extent as those removed by grooming, which was maximal when brood was emerging. Likewise, Kirrane et al. [48] found that mite-fall was positively correlated with VHS behaviour in Russian honeybees. Hence, a considerable proportion of the fallen and the damaged mites could result from VSH or cell cleaning activities (Fig. 1). Moreover, dead mites could also have been damaged by other organisms scavenging in the hive debris, like wax moth larvae or ants. Andino & Hunt [78] showed that grooming activity does correlate with the proportion of fallen damaged mites, but explains only 23% of the variance. Thus, the percentage of damaged mites is not a reliable indicator of the extent of successful grooming taking place.

#### Other methods to measure and quantify grooming behaviour

There are additional methods to assess grooming behaviour in response to *Varroa*: direct records of grooming in observation hives [79], bioassays with frame with several hundreds of bees [78], and bioassays with individual bees or small groups of bees in Petri dishes [64, 80, 81].

Of these, direct observations using an observation hive resembles a natural setting. Also, it measures grooming effort and its success directly, avoiding the problems of interpretation associated with mite fall data. The method is unfortunately time-consuming, which has been the reason for the more common use of simple bioassays with isolated bees in Petri dishes [64]. These bioassays are useful to measure differences in the rate of grooming behaviour between different bee populations. These do not, however, provide a measure of mite mortality. Mites dislodged from the bee’s body by grooming fall on the bottom of the Petri dish and can remount the bee [64]. On a vertical frame, mites dislodged from bees may fall to the bottom of the hive. Simple bio-assays are also not suitable to measure allo-grooming frequency. The frequency of mite body injuries showed no correlation with bees’ auto-grooming capacity [82]. This suggests that most injuries to mites are likely to be caused by an activity other than auto-grooming per se, e.g. during allo-grooming, hunting of non-phoretic individuals or by VSH. Therefore, data obtained by observing isolated bees in Petri dishes, as has been proposed [64] as an assay for grooming, may be un- or weakly related to mite fall and percentage damaged mites in a colony.

Andino & Hunt’s [78] bio-assay with a single frame still uses mite-fall and proportion of damaged mites to assess effective grooming behaviour. However, they exclude VSH as a source of mite-fall by using a frame ﻿with only nectar and pollen and no brood and thus, most fallen mites must have been removed from the adult bees by grooming. Their assay could be a suitable one to measure grooming behaviour, in particular when a variant with a frame with open brood is used. It could be used in a selection programme to increase grooming.

#### The heritability of grooming behaviour

Büchler et al. [83] selected for an increased proportion of damaged fallen mites. Although they found an increase after several generations, the estimated heritability was low (h^2^ < 0.15 [84];). They concluded that the heritability was too low to justify the laborious sample collection and processing in a large-scale selection program. Stanimirovitz [85] also measured heritability as percentage damaged fallen mites and found a variable but rather low heritability (0,16 < h^2^ < 0,42). Moretto et al. [79] using an observation hive to assess the importance of grooming by direct observation, found an estimated heritability of h^2^ = 0.71 + 0.41.

Büchler et al. [84] concluded, against empirical evidence, that the proportion of mutilated mites in the debris of a colony can be used as an indicator of grooming success under field conditions. As only a fraction of the mutilated mites is due to grooming behaviour, their conclusion that the heritability of grooming is too low to justify a selection programme is not supported by proper empirical data. When properly measured, heritability of grooming behaviour could well be high enough for a successful selection programme to increase grooming.

#### Importance of grooming as a defence against Varroa

Grooming against phoretic *Varroa* mites is the only defence that bees have during the long period in winter when there is no brood nest. Honeybee colonies with a high frequency of effective anti-*Varroa* grooming could in this way reduce the weakening and mortality of worker bees during winter and reduce the parasite population to a low level before the new reproductive season starts. Including anti-*Varroa* grooming behaviour in a selection programme for *Varroa*-resistance could therefore be considered, although we do not know if anti-*Varroa* grooming behaviour is an essential trait for resistance against the mite. Andino & Hunt’s [78] bio-assay to estimate grooming frequency would be a good compromise to minimize the laborious sample collection and processing.

### Non-reproducing *Varroa* females: brood effects

#### The hypotheses

Many studies have found evidence that not all female *Varroa* that enter a cell reproduce. There are four hypothesis that can explain non-reproduction of foundress mites:

1. As described above, Harbo and Harris [86] initially interpreted the increased proportion of non-reproduction in the lines selected for *Varroa* resistance as suppression of mite reproduction by the pupae. It turned out that the removal of pupae with reproducing mites by bees from these lines caused most of the increase in the proportion of non-reproducing mites [34].
2. However, non-reproduction can also be a trait of *Varroa*. On its original host, *A. cerana*, a large proportion of adult female mites enter worker cells but do not reproduce and Boot et al. [3] asked why they would enter the worker brood cells if they do not reproduce there. Apparently, reproduction is not the only reason for mites to invade a brood cell. They may invade worker cells of *A. cerana* to hide in safety from the grooming behaviour of adult bees and so survive periods without drone brood [8, 60, 61]. In *A. cerana* the drones are produced during only 3–4 months [9, 10]. With *A. cerana* drone post-capping development times of 13.5–14 days a single fertile mite would have only approximately three to five reproductive cycles per year. This means that adult mites may spend 8–9 months of the year without opportunity to reproduce [4] and would be exposed to grooming behaviour during this time if they would spend it as phoretic mites.
3. A third hypothesis is that there is a constraint on reproduction in these mites This could either be because they have not been inseminated or because of other reproductive problems. Martin et al. [87] summarize the published evidence that in Europe 6–24% of adult *Varroa* females enter cells but do not reproduce. ﻿In an experiment he showed that 8–20% of male *Varroa* offspring in worker cells and 10% in drone cells died before being able to mate. Unfertilized females of *Varroa* only produce male offspring. A recent finding [88] is that unfertilized females may sometimes mate with their own son and then be able to produce daughters during a second reproductive cycle. Yet, unfertilized females can explain a large part of the observed non-reproduction. Other studies found similar results ( [3]: 12% non-reproduction; 11–17% only male offspring). Constrained females may explain most of the non-reproduction of *Varroa* observed in mite-susceptible colonies.
4. A fourth hypothesis deals with *Varroa*-tolerant or resistant bees. Camazine [89] compared *Varroa* reproduction on European Honeybee and Africanized honeybee. He introduced combs of Africanized and European honey bee larvae into mite-infested Africanized bee colonies. In European honeybees, 75% of infested brood cells had female mites that reproduced, while in Africanized honeybees this was only 49%. As only the origin of the brood was different in his experiment, a factor in the brood apparently affected the reproductive success.

Harbo & Harris [90] found that the increase in the proportion of non-reproducing mites by VSH could not explain all non-reproduction. A second trait contributed to this reduction: a genetically based factor from the brood produced by VSH queens reduces mite reproduction [34, 91]. Thus, in these selection lines, VSH is not the only mechanism resulting in a reduction of mite reproduction. Three studies now provide evidence that the mites do not always reproduce after entering a drone cell, and that there are genetic differences between the drones on which mites reproduce and drone brood on which they do not [92–94] (Fig. 1).

The inhibition of *Varroa*’s reproduction by infested pupae, (i.e., a brood effect) is a shared trait of many *Varroa*-resistant *A. mellifera* populations across the globe as well as in the original host *A. cerana* [4, 6, 47, 95–100].

#### The mechanism causing non-reproduction

Stage specific signals of the host larvae initiate and disrupt *Varroa* reproduction [101]. Camazine [89] suggests a lower level of juvenile hormone (=JH) production in Africanized bee larvae as hypothesis to explain the lower proportion of reproduction of *Varroa* in Africanized honeybees. The available evidence published at the time [102, 103] showed that JH titre indeed affects reproduction in *Varroa*. It was hypothesized that this hormone could also regulate oogenesis in *Varroa*, and, in addition, that host-derived JH could be responsible for initiation of reproduction [102]. When more sensitive techniques were available to measure JH titres, follow-up studies [104, 105] could not confirm these results. More recently, Conlon et al. [93] found evidence that a gene from the ecdysone pathway could be involved in the suppression of reproduction of *Varroa*. ﻿*Varroa* requires ecdysone and pupal proteins to initiate vitellogenesis but lacks the genes for ecdysone synthesis [106]. Other chemical signals than ecdysone emitting from the larva could be involved in inducing the *Varroa* mite to enter a cell [107], or, inducing *Varroa* to start reproduction [94]. ﻿Understanding the underlying physiological processes that interfere with the crosstalk between the mite and the host larva will be fundamental to comprehend exactly how the brood effect works [108].

Villa et al. [109] tried to increase suppression of *Varroa* reproduction by selection for the brood effect. They found a significant response during the first two generations of selection but the difference between selected colonies and control colonies disappeared in successive generations. A possible explanation for this finding is that adaptation of mites to host cues occurs in these experiments.

#### Other brood effects

*Varroa*-infested brood from hygienic colonies was more likely to be removed than brood of unselected colonies in cross-fostered brood experiments [110], showing that a factor in the brood is involved in VSH behaviour and mediates *Varroa* resistance. Hence, brood effects and VSH are partly interdependent (Fig. 1). Signal production by parasitized pupae and perception by the adults can both play roles in the detection of infested cells.

### Brood cell size

The natural cell sizes of European-honeybees (*A. mellifera*) were smaller than nowadays found in most bee hives. Beekeepers wanted more productive bees and started to use foundation with larger cell sizes, as this was believed to improve colony performance [111]. Erickson et al. [112], however, suggested that the natural, smaller cell size might be advantageous for a number of reasons, including resistance against *Varroa*. Their hypothesis followed from the observation that Africanized honeybees build small cells (diameter 4.5–4.8 mm) in comparison with those of European bees (diameter 5.1–5.5 mm) [89], and that *Varroa* has a much lower reproductive success in Africanized bees. Independent tests of Erickson et al.’s [112] hypothesis, using a variety of different experimental ﻿designs and a variety of criteria to judge the hypothesis have produced variable results. Heaf [113] provides a review of these studies. He cites 15 studies, of which five provide support for the hypothesis by Erickson et al. [112] hypothesis.

One hypothesis to explain a lower reproductive success of *Varroa* in smaller brood cells is that bees in smaller cells have a shorter developmental time, leaving less time for reproduction of *Varroa*. A second hypothesis is that immature mites may experience difficulty developing in small cells due to lack of space, impeding movement of the mites and possibly causing an increase in mortality of mother mites and offspring*.*

#### Population growth of Varroa

Martin & Kryger [114] found evidence in support of this hypothesis when they compared the number of offspring per cycle of *Varroa* in brood of *A. m. scutellata* with that in brood of the larger *A. m. capensis* bees in *A. m. scutellata* cells. Seeley and Griffin [115] compared bees of the same origin that were either placed on frames with small (4,8 mm) or large (5,4 mm) cells. They ﻿measured population development of *Varroa* once a month—from mid-June to mid-October and did not find differences in population growth of the mites. They attributed the lack of differences to the small difference in available space between the two types of cells, caused by differences in size of bees developing in small and large cells. Likewise, no larger *Varroa* populations were found in hives with large cells [116–119].

#### Number of offspring per cycle

Although in choice experiments smaller brood cells have a smaller probability of being colonized by *Varroa* foundresses [117–120], no effect of cell size on the number of female *Varroa* offspring was found.

Hence, neither the hypothesis that shorter developmental time of bees in small cells results in slower populations growth of *Varroa*, nor for the hypothesis that lack of space in small cells restricts *Varroa* reproduction is supported by experimental results. All the evidence that small cells reduce *Varroa* populations growth come from experiments with African or Africanized bees [114, 116, 120, 121]. Maggi et al. [120] found that *Varroa* was more often non-reproductive in cells with a smaller width. This suggests that the effect is caused by an interaction between cell size and another resistance trait, as explained in the following paragraph.

#### Interaction between cell size and VSH

A possible explanation for the variable outcome of studies on small cell size is an interaction between cell size and VSH behaviour. Smaller cells may ﻿ enhance brood signalling, i.e. in smaller cells suppression of *Varroa* reproduction by the worker larva, or recognition of cells with reproducing *Varroa* could be easier. Evidence for this hypothesis comes from [122]. Some of the selection lines that were used did not show the VSH-trait, and provide evidence that cell-size per se does not influence *Varroa* population growth. In selection lines with the VSH trait the rate of the VSH cleaning behaviour was higher on the smaller cell size. This confirms earlier findings [123]. Hence, the variable outcome of studies on small cell size could be caused by variation in VSH behaviour of the bees used in the different studies (Fig. 1).

### Resistance and tolerance against the *Varroa*-associated viruses

An important reason why *Varroa*-infested colonies collapse is ﻿that *Varroa* activates an infection by Deformed Wing Virus (DWV) and increases the titre of other viruses such as Sachbrood virus and Black Queen cell viruses. The latter two have also been implicated in *Varroa*-induced bee mortality [124].

These viruses have been associated with the death of millions of European honey bee colonies across the world. Bees in the absence of such viruses can sustain much larger *Varroa* populations before collapse than bees exposed to the virulent forms of these viruses, as was observed in South Africa [125]. The interaction of *Varroa* with the DWV–honeybee interaction somehow resulted in the virus becoming much more virulent [126], although this has not happened in the honeybee population on the island of ﻿Fernando de Noronha in Brazil, [126]. *Varroa* surviving bees in the Swindon honeybee conservation project also were predominantly infected with an a-virulent type of DWV [127]. Vertical transmission favours the evolution of lower virulence [128, 129]. As vertical transmission is more frequent in closed populations, it is expected that dominance of the avirulent form of DWV is found on islands, as Fernando de Noronha [126], or otherwise isolated populations, like Gotland [129], Arnot forest [128], Swindon [127]. In addition to lower virulence of the parasite, increased tolerance/resistance of the host is also favoured in a system with predominantly vertical transmission. Evidence for the role of virus tolerance in the Gotland population comes from [130–132] and [133]. Likewise, *A. m. scutellata* seems resistant or tolerant to DWV [134].

The evolution of lower virulence in DWV and other viruses is also favoured by VSH. The hygienic bees preferentially target pupae that have been damaged by a virulent form of the virus [135], and so can be instrumental in making a less virulent type dominant (Fig. 1).

### Bee life-history traits that may hamper *Varroa* population growth

Colony size and swarming frequency are life-history traits of bees that affect population growth of *Varroa.* Although these traits are to some extent heritable, they are largely determined by environmental factors like nest size and food abundance.

The mechanisms whereby smaller and more frequently swarming colonies have greater resistance to *V. destructor* include having relatively few brood cells, especially drone brood, which limits reproductive possibilities for the mites. Frequent swarming also helps control the mites because a swarming event exports about 35% of a colony’s mites [136]. Furthermore, swarming temporarily deprives the mites of brood, and its absence disrupts the mites’ reproduction and increases their exposure to grooming. Feral bees in Arnot Forest N.Y. were infested with *Varroa* mites but had a stable population size with established colonies having a lifespan of 5–6 years [128]. Seeley [137] and Loftus et al. [136] tested the hypothesis that persistence of these wild colonies is at least partly due to their habit of nesting in small cavities and swarming frequently by comparing colonies in small and large hives. These results confirm that smaller nest cavities and more frequent swarming of wild colonies contribute to their persistence without mite treatments. In addition, Seeley and Smith [138] showed that crowding of beehives in apiaries ﻿increased *Varroa* transmission between colonies. They concluded that the scattered distribution of wild colonies makes them less exposed to horizontal infection from other colonies by drifting and robbing as occurs in apiaries [139]. This also contributes to the persistence of these colonies.

### Honey bee mating system and population structure and natural selection

In eusocial insects there is strong selection for increased genotypic diversity in worker offspring to either meet the demands of different tasks or to mitigate the effects of parasitism. Although all workers in a bee colony stem from a single mother, i.e. the queen, genetic variation among workers can be increased by two mechanisms. One of them is to increase the rate of recombination, the other is by polyandry. Indeed, the rate of recombination in honeybees is among the highest measured in the animal kingdom [140] and honeybee queens are extremely promiscuous. ﻿There is strong evidence that the genetic diversity that a polyandrous queen generates in her colony benefits its members by enhancing workforce productivity [141–143]. As honeybees have single locus sex determination, the high genetic diversity is also important to prevent homozygosity in the sex-alleles, which results in non-viable diploid males instead of worker bees or queens.

Honeybee queens mate in flight [144] with 7–28 drones [145, 146], which may originate from colonies up to 15 km away [147]. Young virgin queens leave the colony for a mating flight. In honey bees the mating system is characterized by “drone congregation areas” (DCA’s) that are visited by males from many colonies. Baudry et al. [148] estimated that the drone aggregation area they studied attracted drones from 238 different colonies. Jaffé et al. [149] found lower numbers in a study of *A. m. scutellata* in South Africa. The number of colonies from which drones arrive in a particular DCA depend on the density of colonies in the area and on the presence or absence of physical barriers (e.g. bodies of water or mountains). Given the high promiscuity of queens, the long distances travelled by queens and drones and the large numbers of colonies from which drones in a DCA originate, the conclusion must be that honeybees have a panmictic population structure. This mating structure is important for how natural selection or selective breeding for *Varroa* resistance proceeds when queens are allowed to mate freely. While selective breeding and natural selection favour resistance traits, mating of young queens with drones from the surrounding populations counteracts the effects of local selection.

### Natural selection for *Varroa* resistance: the evidence

As documented in the first part of this review, *A. mellifera* possesses a number of heritable traits that contribute to its resistance against *Varroa*. Given the high mortality that *Varroa* inflicts on non-resistant colonies, it is expected that natural selection upon invasion by *Varroa* would quickly select for increased frequencies of the resistance traits.

### Resistance of a. m. Scutellata and A. m. capensis in South Africa

*Varroa* was discovered in South Africa in 1997, where it was most likely introduced with ﻿a commercial transport of bees and queens. Two sub-species of honeybee are found in South Africa: the Cape honeybee (*A. m. capensis*), a coastal race occurring in the fynbos biome along the southwest and south coasts of South Africa [150], and the Savanna honeybee (*A. m. scutellata*) in the rest of South Africa. The mite spread rapidly and after five years was found throughout the country. During peak infestations on average 10,000–17,000 mites could be found in a single colony, and sometimes even 30,000–50,000 mites [125]. Evidently, there was no immediate impediment to *Varroa* mite reproduction in African Cape and Savanna honeybee colonies and the mite was able to reproduce very efficiently in in both bee subspecies, at least initially. At the peak of the infestation 30% of colonies collapsed. There was, however, no population-wide collapse of colonies and the majority survived [125]. ﻿The tolerance of Cape and Savanna honeybees for higher infestation rates is likely to be due to the absence of deleterious virus outbreaks (e.g. by a virulent form of deformed wing virus) in the South African bees [134]. Although a number of bee viruses have been found in South and East Africa [151, 152], and Cape honeybee pupae and adults were found to be susceptible to virus infections, it was not possible to induce any bee viruses from Cape honeybee colonies, suggesting a general absence of virulent bee viruses in this population [125].

After the peak infestation mite densities gradually decreased and Cape honeybees (*A. m. capensis*) became resistant 3–5 years after the arrival of *Varroa*, while Savannah honeybees (*A. m. scutellata*) became resistant after 6–7 years [125]. To date, *Varroa* is no longer a problem in South Africa: in Mike Allsopp’s words, “*Now, it is no more than an arbitrary presence”.*

Important for the evolution of resistance against *Varroa* in South Africa was that the original recommendation given to beekeepers that no chemical treatment should be used until it had been ascertained that *Varroa* would result in honeybee colony collapse. An additional reason why natural selection could work swiftly was the presence of a large wild honeybee population. Hence, natural selection could operate in both commercial and wild bees, unhampered by the widespread use of acaricides.

As only a relatively minor part of the bee population collapsed, mortality by *Varroa* did not cause a genetic bottleneck that would otherwise have hampered the evolution of resistance.

The following traits have been invoked to explain the resistance (see also Table 1).

1. Hygienic behaviour [47, 153, 154]. Fries & Raina [155] report that 77% of pin-killed brood is removed by *A. m. scutellata* in 24 h, a removal rate much higher than reported for European bees [29, 153, 156]
2. Grooming: [154], measured as the percentage of damaged fallen mites
3. Non-reproduction: [98, 114]. ﻿Reproductive failure has increased over time [157], suggesting that VSH and or a brood effect has increased over time.
4. Short developmental time. Allsopp [125]﻿ concluded that the shorter post-capping stage (between 9.6 and 12 days in Cape honeybees, 10–12 days in *A. m. scutellata*, [114, 158, 159] can reduce reproductive output of *Varroa.*

**Table 1:** Traits that have been measured and/or invoked to explain resistance

Populations	*A. scutellata* & *A. capensis*	Africanized bees in South America	Arnot Forest, New York	Primorsky	Gotland Sweden
Traits					
Hygienic Behaviour	yes	yes	No evidence	yes	no
VSH	yes	yes	No evidence Yes	yes	no
Grooming	yes	yes	No evidence Yes	yes	no
Non-reproduction	yes	yes	No evidence	yes	yes
Shorter Developmental time	yes	yes	no	no	no
Virus tolerance or resistance	possibly	possibly	Possibly, No evidence	no	Yes
Life-history & spatial distribution of colonies	no	no	yes	yes	no

VSH has never properly been measured in South Africa, but is an important trait in the resistance of a Kenyan population of *A. m. scutellata* [47]. Moreover, it plays an important role in Africanized bees in South America [158].

The most plausible way to explain the changes in abundance of *Varroa* after its arrival in South Africa is that the frequency of resistance genes in the bee populations of *A .M. scutellata* and *A. m. capensis* were too low to prevent the observed population explosion, but that most colonies survived peak densities of *Varroa* because they could tolerate high densities in the absence of virulent viruses that otherwise would have destroyed the colonies. Natural selection for resistance against the mites subsequently took over, resulting in an overall low *Varroa* density.

It is likely that the alleles for the resistance traits i.e. hygienic behaviour and grooming were already present at frequencies higher than found in European honeybees, before *Varroa* colonized Africa, as Africanized bees showed these behaviours when *Varroa* arrived in South America.

### Resistance of Africanized bees

In 1957 African *A. m. scutellata* bees imported from Tanzania into Brazil to improve honey production in tropical South America escaped from an experimental apiary and hybridized with European bees. The hybrids spread in South America and colonized Central America and the southern United States.

Although African honeybees came to Brazil long before *Varroa* colonized Africa and before the African *A. m. scutellata* bees had developed resistance against the mite, the *A. m. scutellata* hybrids showed already tolerance or resistance to *Varroa.* Soon after the discovery of *Varroa* in 1979, the levels of infestations detected were a source of concern for Brazilian apiculture, although there were no reports of bee colony deaths [160]. It soon became clear that Africanized bees can survive *Varroa* infestation without treatment [153, 158, 161–163]. Rapid natural selection seems to have resulted in increased resistance and treatment against *Varroa* is generally not practiced. ﻿Losses of Africanized honey bee colonies due to varroosis are not reported and possible negative effects on honey production seem to be negligible [160]. This is surprising, as in contrast to South Africa, viruses associated with *Varroa* like Deformed Wing Virus are widespread in South America [164–166] and Africanized bees are not resistant against the virus [167, 168], although in one study, the rate of virus increase was lower in Africanized bees than in European bees [169].

The Africanized bee is the common race of honeybee in Brazil. An important prerequisite for the rapid evolution of *Varroa* resistance was the enormous number of feral colonies of Africanized honey bees in Brazil. Even in natural rainforest ecosystems without any beekeeping activities, the honey bee is the predominant pollinator. Obviously, colonies managed by beekeepers represent only a small percentage of Brazil’s honey bee population. Therefore, the feral honey bee population is permanently exposed to selection for *Varroa* resistance. ﻿In Mexico, the Africanized honeybee was established for the first time in 1992 and was found to be resistant against *Varroa* already in 1994. Thus, it appears unlikely that the resistance evolved there and more likely that the bees invading from Brazil were already resistant [170].

The following traits have been invoked to explain the resistance (see also Table 1):

1. Hygienic behaviour: Africanized bees are generally regarded as having better hygienic behaviour than European bees [51, 68, 70]. found only slightly better hygienic behaviour in Africanized bees, most likely because they worked with artificially recapped cells.
2. VSH: [46, 171, 172] and provide experimental evidence that Africanized bees have a higher rate of VHS behaviour than European bees. Although the first two publications precede Harris’ [37] publication in which VSH was defined, the behaviour described in these papers is clearly VSH.
3. Grooming: Africanized bees are more efficient in removing phoretic *Varroa* by grooming than European honeybees [68, 79, 171].
4. Non-reproduction: *Varroa* shows a higher rate of non-reproduction in the Africanized honeybee from Brazil and Mexico, than in European honeybees [171, 173]
5. Shorter developmental time: post-capping period 11.5–11.6 days in Africanised honeybees compared to 11.6–12.0 days in European honeybees [174]

#### Survival of feral honeybees in Arnot Forest in New York USA

*Varroa* arrived in the U. S in the mid-1980s. ﻿A feral population of bees breeding in hollow trees had been censused in 1978, prior to the introduction of *V. destructor* to North America [175]. ﻿The census was repeated in 2002 when *Varroa* had established itself [128]. The number of bee colonies in the forest had not changed. Swarms from this feral population were trapped in the forest and placed in hives to study if the colonies suppressed the *Varroa* mites. In 2005, no differences in mite population growth were found between the feral bees and commercial non-resistant *A. m. carnica* bees. It is possible that the relatively short period of time during which the bees of the Arnot Forest had been exposed to *Varroa* is the reason that alleles for resistance traits had not yet increased, but that the bees persisted because of small colony sizes [138], frequent swarming and the widely spaced colonies [137]. An untested additional hypothesis is that mites and/or the viruses vectored by them may had become less virulent. When colonies are widely separated, their parasites and pathogens are probably transmitted mostly vertically (from parent colony to offspring colony) through swarming, a scenario that selects for decreased virulence [128]. Another possibility is that the Arnot Forest bees were *Varroa* tolerant because they had evolved tolerance or resistance against the associated viruses, as was found for a Swedish population [130]. Ten years later, genomic data showed that mayor genetic changes had occurred since 1978 [176] and convincing evidence for *Varroa*-resistance in the Arnot Forest honeybee population was found [177]: the feral bees from Arnot Forest are able to reduce mite populations by increased grooming and VSH behaviour.

#### Selection for resistance in Prymorski bees

European settlers took *A. mellifera* (spp. *caucasica & carnica*) in 1865 to far eastern Russia (Primorsky) [178, 179]. The area has native *A. cerana* infested with *V. destructor* which most likely infested the arriving *A. mellifera*, resulting in the longest known association of *A. mellifera* and *V. destructor* [1, 180]). Preliminary examinations of *A. mellifera* in the Primorsky territory suggested that they might have substantial levels of mite resistance [180]. These observations inspired the importation of 362 *preselected* queens into North America from 1997 to 2002 [181] for further testing of these Primorsky honey bee queens and the start of a selection programme in the USA for *Varroa* resistance by colony-level selection [182]. An initial evaluation indicated that their commercial traits such as honey production were similar to those of existing commercial stocks [183]. Most importantly, some of the imported Primorsky queens produced colonies which appeared to be resistant to *V. destructor* [183]. After eight years of selection several Primorsky queen lines show mite population growth < 1 and thus were *Varroa* resistant [184]. The other lines had growing *Varroa* populations and some of them did not much better than commercial Italian bees. Further selection decreased the variance between lines and resulted in overall resistance in the Russian bees [58, 185]. The resistant Primorsky bees exhibited strong grooming traits [1], high hygienic behaviour, VSH [48], reduced brood attractiveness for *Varroa*, and decreased reproductive success of *Varroa* in combs built by the Russian honeybees [186] (See also Table 1). Unlike Italian colonies they either slow down or completely stop brood production in response to a lack of nectar flow [187]. This resource sensitivity may also contribute to Russian honey bees’ *Varroa* resistance by interrupting *Varroa* reproduction.

Hence natural selection in eastern Russia had resulted in a high but variable presence of resistance traits. Artificial selection at colony level in the US in isolated mating yards resulted in fully resistant Russian bees. The use of isolated mating yards has prevented the loss of resistance alleles by the dilution effect of the panmictic mating structure, and a well-designed breeding schedule prevented the loss of genetic variation.

#### Resistance in European bees

﻿﻿Unlike *A. m. scutellata and A. m. capensis* in Africa and the *A.m. scutellata* hybrids in South America, European subspecies of bees suffered massive colony collapse upon the arrival of *Varroa* from Russia. The new parasite devastated natural populations of *A.mellifera* in Europe and feral populations in North America, and beekeepers experienced massive mortality of colonies. They had no other choice than using acaricides, organic acids or essential oils to kill the mites, thus hampering natural selection for resistance. Other apicultural practices that are unfavourable for the evolution of *Varroa* resistance are: crowding together of colonies in apiaries, so that horizontal transmission of *Varroa* is favoured; managing colonies to be unnaturally large, so that they have high honey production and low swarming rates; moving colonies from place to place, so that there is strong gene flow that prevents natural selection from altering locally adaptive allele frequencies in a closed population; and regular re-queening of colonies with pure-bred non-resistant queens. All these practices contribute to making apiaries an ideal environment for *Varroa* mites and the viruses they vector.

In Europe and North America, resistance traits are present, albeit at a low frequency in the population and their expression inside colonies is reduced by the bees in a colony that do not have the resistance alleles [188]. The presence of resistance alleles has been shown by efforts to select for increased hygienic behaviour [24], grooming [73], VSH and suppressed mite reproduction [110]. However, the expression of resistance alleles in the European honeybee populations is not strong enough to prevent the *Varroa* population to grow and to prevent colonies from collapse. Natural populations of *A. mellifera*, and the pollination afforded by them, have largely been eliminated by the mite in Europe [100], threatening the majority of the ten European subspecies with extinction and making that natural selection for *Varroa* resistance cannot proceed in populations not submitted to apicultural practices.

Yet, a number of European studies discovered colonies and populations of honeybees that survived the invasion of *Varroa* without treatment against the mites (Table 1). Surviving bees were found in Yugoslavia [189]. Starting with three colonies that survived an epizootic of *V*. *destructor*, a stock of honey bees was produced which was only slightly more resistant than other stocks. Surviving populations were also found in France [190] and in Norway [99]. Other studies report the importation of bees known to be already *Varroa*-resistant and their survival without treatment after importation and hybridisation with local bees: *A. m. intermissa* imported from Tunisia [191, 192] and surviving bees from Gotland, Sweden [81, 193] for one of their populations.

In addition, there is one well-documented large scale experiment with a population of untreated colonies kept without treatment [129]. These studies claim that sometimes local conditions allow the evolution of *Varroa* resistance by natural selection. In the following we will explore why evidence for the evolution of resistance in *Varroa* in Europe is rather scanty.

Fries et al. [129] founded a genetically diverse honey bee population of 150 colonies on a peninsula at the southern tip of the island Gotland in the Baltic sea, isolated from the main island through a narrow land bridge. Swarms produced were added as new colonies to the population. After four years, 38 new colonies had been established from swarms, but mortality due to *Varroa* infestation resulted in only 13 colonies of the 188 surviving after four years. The colonies surviving after four years had mite infestations that were more than halved in comparison with the third year, before massive colony collapse occurred. In addition, surviving colonies had fewer worker-bees and produced fewer drones [194, 195]. The small number of colonies surviving resulted in a genetic bottleneck and strong inbreeding [107]. The experiment shows that genetic variation for resistance was present in the population before selection and that natural selection to improve *Varroa* resistance is possible in closed populations, albeit at a price of lower brood production.

Le Conte et al. [190] collected 82 colonies that had survived the invasion by *Varroa* without treatment. They were placed in the region where they had been found: 30 in an apiary near Le Mans, and 52 in an apiary in Avignon. Treated control colonies were placed nearby. The mortality of colonies varied between 9.7 and 16.8% per year. Surviving colonies had only 32.4% of the *Varroa*- infestation rate in control colonies. Honey production by untreated surviving colonies was half of that of the controls. The experiment shows that the collected colonies had some degree of *Varroa* resistance at the start of the study. The surviving colonies were maintained under artificial selection as is witnessed by this citation [196]*:“What has happened to these bees since we published those results in 2007? Once every two years, we graft queen larvae from the three best colonies in each apiary (west and south of France) to get 20 colonies. The queens are naturally mated by local drones. About 30–35% of the colonies die within 18 months, but the rest of the colonies are good candidates for surviving to the mite, so the stock still survives efficiently”*. Hence, despite continued selection, *Varroa* resistance has not increased over a 10-year period.

The natural and artificial selection for colony survival did not increase the frequency of resistance genes probably because the bees are kept in an open panmictic breeding population. While selection favours colonies with a higher frequency of resistance alleles, panmictic mating in a population with a low frequency of these alleles makes that queens of the selected colonies mate with drones with a low frequency of the resistance alleles, In addition, drones with resistance genes from the untreated colonies disperse into the environment. Thus, the mating structure of the population counteracts local selection for *Varroa* resistance.

Colonies descending from both the Avignon and Gotland survivor populations both still harboured growing mite populations with more than 0.7 fertile female offspring per foundress per cycle, and would have collapsed if left to natural selection alone [162]. They survived because they were subjected to prolonged artificial selection and periodically multiplied by breeding a large number of queens from the best colonies [194, 196]. When the Avignon bees were tested outside their native environment in a Europe-wide experiment [197], neither their *Varroa* infestation rate after one year without treatment nor their survival outperformed that of colonies descending from non-selected genotypes tested at the same locations.

The surviving colonies from the Østlandet region in Norway were part of an open panmictic breeding population (“the population being within sufficient distance of known susceptible colonies from various backgrounds (mostly *A. m. mellifera*, *A. m. carnica*, Buckfast) that would facilitate horizontal parasite transfer” [99]). These colonies were also multiplied by splitting the healthy surviving ones to replace lost colonies [99] and hence they were also under a continuing regime of artificial selection. The surviving colonies harbour growing mite populations (0.87 fertile female offspring per foundress).

Which mechanisms are involved in reducing mite reproduction in these populations? The role of hygienic behaviour was investigated in the surviving Gotland colonies by studying the fate of 100 pin-killed pupae [195]. Removal rate in 12 h was only 15% and not different from the 20% found in control colonies, not supporting a role for hygienic behaviour. Unfortunately, the authors did not test for VSH behaviour, leaving the possibility that the removal of mite infested pupae plays a role in reducing *Varroa* reproduction. They also measured the percentage of damaged mites of mites fallen onto the bottom board. As we have seen above damaged mites can both result from VSH behaviour as well as from grooming behaviour. They found 31–35% of damaged mites, which was not different from the percentage found in unrelated control colonies. Although the results are not supporting the hypothesis that grooming plays a role in the reduction of the *Varroa* population, they cannot be taken as evidence that grooming plays no role. Experiments using a better assay to assess grooming, e.g. that of [78] are needed. The surviving Gotland colonies had a significantly lower proportion of mites that reproduced successfully (48% versus 78% in control colonies) [195]. Failure to reproduce resulted from ﻿infertility, absence of male offspring, high proportion of mite offspring mortality, or delayed egg-laying by the mother mite. As we have seen, there are four possible mechanisms to explain an increased percentage of non-reproduction in *Varroa,* two of which caused by the bees, i.e. VSH behaviour and a brood effect. Proper experiments on VSH behaviour and a brood effect are needed to distinguish between these hypotheses. As the resistant bee populations originated from only 13 surviving colonies, it is possible that traits like VSH and grooming were not present in the small sample. Locke and Fries [195] suggested that the smaller colony size of the surviving bees is an adaptation that would reduce reproductive rate of *Varroa*. As smaller colony size decreases colony fitness in many ways, we prefer the alternative hypothesis that the smaller colony size is non-adaptive and an effect of inbreeding in this population.

They also suggested that the Gotland bees were resistant to viruses that normally cause colony collapse. This was confirmed by [130–132] and [133]. Why did the Gotland experiment not result in fully resistant bees? As only 13 of 188 colonies survived, and ﻿genetic drift had caused an extreme loss of genetic diversity in the surviving population, it seems likely that insufficient genetic variation hampers the evolution to full resistance in this population. This hypothesis should be tested by increasing the genetic variation in this population and document the changes in mite reproductive success.

The *Varroa* mites in colonies of the Avignon population of surviving bees also had a lower proportion of reproducing mites, with non-reproduction being the most important factor [198]. The mechanism causing the reduction in reproduction was not determined. VSH could possibly explain the supressed mite reproduction in this population, as genetic evidence suggests that the suppressed mite reproduction is caused by a behavioural trait [199]. The mechanism of resistance in the Le Mans population has not been studied (See also Table 1).

Reduced reproductive success of *Varroa* was also observed in the surviving colonies from the Østlandet region in Norway [99]. This cannot be completely explained by VSH behaviour as the frequency of VSH was only 5%. Oddie et al. [200] suggested that a slightly shorter post-capping period for the brood of surviving colonies in comparison with non-related controls could have contributed to the lower *Varroa* reproductive rate, but did not calculate whether this small difference could produce the observed effect on mite reproduction, which seems unlikely. Other mechanisms, like a brood effect were not studied.

Kefuss [191, 192] started *Varroa*-resistant lines by importing *A.m. intermissa* from Tunisia. Judging from the very high rates (40–75%) of non-reproduction in these bees they had already a high incidence of VSH behaviour when arriving in France. Evidence for high rates of VSH and grooming in these bees is provided by [62, 201]. In a Dutch experiment on survival of untreated bee colonies Panziera et al. [193] found evidence for the role of VSH in reducing mite populations.

None of the European studies cited above have resulted in a fully *Varroa*-resistant population. This is either because surviving colonies were part of a panmictic population and surrounded by colonies with low frequency of resistance traits, or, in the only example of a closed population (Gotland), because of inbreeding.

## Conclusions

The host shift of *Varroa destructor* to *Apis mellifera* and the fast colonization of *A. mellifera* populations in Europe, the Americas and Africa initially resulted in considerable mortality exerting strong selection for the evolution of resistance against the mites. While selection resulted in resistance in populations of *A. mellifera scutellata* and *A. m. capensis* in Africa and in *A. m. scutellata* hybrids in South America, resistance did not evolve in honeybees in Europe and North America. Most unprotected colonies of European honeybees in these continents succumb when infested with *Varroa* and resistance to *Varroa* has not increased in the 40 years after the *Varroa* invasion.

We have reviewed research on traits for *Varroa* resistance and the literature that documents the evolution of resistance to *Varroa* in Africa and in South America. We have also reviewed studies of several populations on honeybees in Europe and North America that were not treated against *Varroa* and the evidence these studies provide for natural selection of *Varroa* resistance.

From reviewing this evidence, we conclude that:

1. Natural selection can result in population-wide resistance in large panmictic populations only when a large proportion of the population survives the initial invasion of *Varroa*. This is what happened in Africa and South America.
2. When, upon invasion of *Varroa* a major part of the bee population collapses or is protected against the mite by chemical treatment, natural selection for resistance does not succeed. This is what occurred in Europe and North America.
3. Small colony size, frequent swarming and widely spaced colonies of wild or feral honeybees reduce the risk of colony collapse due to *Varroa* infestation. These life-history and ecological characters promote vertical transmission of mites and viruses are therefore conducive to the evolution of less virulent mites and viruses and more resistant bees. These characters also allow the evolution of resistance in wild bee populations. This is shown by the study of the bees in Arnot Forest.
4. The panmictic mating structure of honey bees prevents local natural selection for resistance, because resistance genes disperse into neighbouring populations at a rate higher or equal to the local rate of recruitment of these genes by selection.
5. Selection by breeding can increase the level of resistance of colonies and so increase the proportion of resistant colonies in the population as whole. When this proportion is high enough, bee keepers can stop chemical treatment and natural selection can proceed.
6. In closed populations, as on islands, natural selection is not counteracted by the dispersal of resistance genes, and natural selection can proceed, unless it is constrained by inbreeding. This is what the Gotland experiment has shown.

In most of the populations that developed resistance against *Varroa*, behavioural defences against the mites are important: grooming against phoretic mites and hygienic behaviour, or more precisely VSH against reproducing mites. In addition, brood effects and shorter developmental times play a role in reducing *Varroa* mite reproductive success. The exception is the Gotland experiment, in which no evidence was found for grooming or hygienic behaviour (Table 1). On the whole these results show that natural selection favours traits that also have been used in selective breeding programmes.

The ultimate goal of making European and North American honeybees resistant against *Varroa* is within reach. Artificial selection using single drone insemination as pioneered by [32] and [86] can be used to increase the frequency of resistance alleles in the honeybee populations of both continents. Natural and artificial selection at colony level can also be used in closed populations (e.g. on islands or otherwise isolated mating yards) providing that genetic variation in these populations is maintained. Resistant colonies produced in this way can then be used to increase the level of resistance in large panmictic populations. Once the resistance level has passed the threshold where it becomes profitable for apiculturists to stop chemical treatments of the mite, natural selection can proceed to make European and North American honeybees fully *Varroa* resistant.

## Data Availability

Not applicable.
