# Correction to: Natural selection, selective breeding, and the evolution of resistance of honeybees (*Apis mellifera)* against *Varroa*

**DOI:** 10.1186/s40851-020-00162-8

**Published:** 2020-06-15

**Authors:** Jacques J. M. van Alphen, Bart Jan Fernhout

**Affiliations:** 1 Naturalis Biodiversity Centre, 2333 CR Leiden, The Netherlands; 2 Arista Bee Research Foundation, Nachtegaal 2, 5831 WL Boxmeer, The Netherlands

**Correction to: Zoological Lett (2020) 6:6**

**https://doi.org/10.1186/s40851-020-00158-4**

Following publication of the original article [1], the authors reported an error in Table 1 of the article: there were two instances of “No evidence Yes” in the fourth column, where it should read “yes”.

**Table 1:** Traits that have been measured and/or invoked to explain resistance

Populations	***A. scutellata*** & ***A. capensis***	Africanized bees in South America	Arnot Forest, New York	Primorsky	Gotland Sweden
Traits					
Hygienic Behaviour	yes	yes	No evidence	yes	no
VSH	yes	yes	yes	yes	no
Grooming	yes	yes	yes	yes	no
Non-reproduction	yes	yes	No evidence	yes	yes
Shorter Developmental time	yes	yes	no	no	no
Virus tolerance or resistance	possibly	possibly	Possibly, No evidence	no	Yes
Life-history & spatial distribution of colonies	no	no	yes	yes	no

The original article [1] has been updated to correct this.

Please also find the corrected version of Table 1 in this article for reference.

## References

[1] van Alphen JJM, Fernhout BJ (2020). Natural selection, selective breeding, and the evolution of resistance of honeybees (*Apis mellifera)* against *Varroa*. Zoological Lett.

